# Long‐COVID in people with intellectual disabilities: A call for research of a neglected area

**DOI:** 10.1111/bld.12499

**Published:** 2022-08-29

**Authors:** Gregg H. Rawlings, Nigel Beail

**Affiliations:** ^1^ Psychology Department Nottingham Trent University Nottingham UK; ^2^ Clinical Psychology Unit University of Sheffield Sheffield UK

**Keywords:** coronavirus, COVID‐19, inequality, learning disability, pandemic, post‐COVID syndrome

## Abstract

**Background:**

Long‐COVID (also known as post‐coronavirus‐19 syndrome) is a term used to describe symptoms that people experience following their recovery from the COVID‐19 virus. The severity of long‐COVID is well recognised, with healthcare providers commissioning services to diagnose and treat those affected, as well as funded research into the condition.

**Methods:**

We performed a systematic search for relevant articles but were unable to find any research on long‐COVID in people with intellectual disabilities. Due to the lack of data, we have only been able to make extrapolations from what is known about the condition within the general population.

**Findings:**

We provide an overview of long‐COVID and its potential relevance to people with an intellectual disability. We have focused specifically on symptoms or signs, prevalence, risk factors and treatments of the condition in this group, highlighting areas for clinical practice and future research from a psychosocial perspective. We raise serious questions about our current understanding and the availability of the evidence‐based to inform treatments tailored towards this population.

**Conclusion:**

This is the first report that we are aware of on the topic of long‐COVID in people with an intellectual disability. The lack of research is preventing us from gaining a greater understanding of how the condition impacts people with an intellectual disability.

## Accessible summary

- People with an intellectual disability have been disproportionately affected by the COVID‐19 pandemic.
- Long‐COVID is a condition in which people continue to experience symptoms after recovering from the COVID‐19 virus.
- We have been unable to find any research on long‐COVID in people with an intellectual disability.
- It is not clear what evidence is currently guiding treatments for long‐COVID in this population.
- More research is needed to investigate the rate, symptoms, risk factors and treatments for long‐COVID in people with an intellectual disability.

## 1 INTRODUCTION

It was recognised early in the COVID‐19 pandemic that people with an intellectual disability may be disproportionately affected by this virus. Indeed, healthcare services as well as governments imposed specific measures to best support this group of individuals (Department of Health and Social Care, 2020). Such efforts were established—in part—to protect this group from the virus, reduce the subsequent impact of infection‐control procedures, maintain the provision of services and support, and due to a real fear that the pandemic would further contribute to the inequalities that people with an intellectual disability commonly experience (Rawlings et al., 2021).

Sadly, these fears have been realised (Courtenay & Cooper, 2021; Epstein et al., 2021; Linehan et al., 2022) with reports showing COVID‐19 mortality rates tend to be higher for this group (Williamson et al., 2021). For example, in England (United Kingdom), rates were 2.3 times higher than that seen in the general population (Public Health England, 2020). Similar results have been observed in a study involving over 64 million individuals living in the United States, which found having an intellectual disability was one of the strongest independent risk factors for presenting with COVID‐19 and COVID‐19‐related mortality (Gleason et al., 2021). Rates of comorbidity have also been high in this group (Lunsky et al., 2022)—as well as in their caregivers (Bailey et al., 2021; Sheerin et al., 2022)—as difficulties such as anxiety, stress, sadness, anger, loss and loneliness have been reported.

We have now passed the anniversary of unprecedented nationwide lockdowns announced 2 years ago across the United Kingdom (Prime Minister's Office 10 Downing Street, 2020) and many other nations around the world. However, there is no denying that we are still living with the lasting impact of the pandemic. This has also been highlighted by long‐COVID (also known as post‐COVID‐19 syndrome), which is the term that has been coined to describe the prolonged set of symptoms that people may experience following their recovery from the COVID‐19 virus (SARS‐CoV‐2 infection). Indeed, some authors have suggested long‐COVID may pose another public health crisis, following the impact of the initial pandemic (Garg et al., 2021). Three separate phases of the COVID‐19 virus have now been identified: ‘acute COVID‐19’ characterised by symptoms of the virus lasting up to 4 weeks; ‘ongoing symptomatic COVID‐19’, where symptoms can be experienced between 4 and 12 weeks following the acute phase; and ‘post‐COVID‐19 syndrome’ with symptoms lasting 12 weeks or longer and that are not explained by alternative diagnoses (National Institute for Health and Care Excellence, Scottish Intercollegiate Guidelines Network & Royal College of General Practitioners, 2022). The severity of long‐COVID is now well recognised, with NHS England and NHS Improvement (2021) proposing a five‐point plan for long‐COVID support, which focuses on developing clinical guidelines, investing in assessment and treatments including setting up specific services across England, and funding much needed research.

Throughout the pandemic, the authors have been aware of the impact on people who have intellectual disability and on their access to community learning disability health services—we have published a series of studies of our service's responses (Purrington & Beail, 2021; Purrington & Nye, & Beail, 2021; Rawlings et al., 2021). However, we have become concerned about the lack of data examining long‐COVID in people with an intellectual disability, which was also recognised over 1 year ago in an hour‐long podcast by the Association of Healthcare Philanthropy focusing on long‐COVID and intellectual (learning) disabilities (AHP Leader, 2021), with over 600 views (at time of writing). With the aim of identifying studies to inform this article and help validate our argument that as of yet this is a neglected area, we performed a systematic search of four databases [Web of Science (*n* = 74), PubMed (*n* = 42), PsycINFO (*n* = 33) and Cochrane Library (*n* = 5)], using the search terms: long COVID* OR ongoing symptomatic COVID* OR post? COVID* syndrome AND intellectual disabilit* OR learning disabilit* and the criteria of being published after November 2019 [it is believed this is when the first human was infected by the virus (Tichopád et al., 2021)]. Screening of the title and abstract of the 154 articles identified, followed by a review of the full text, revealed that none of the articles focused specifically on long‐COVID in adults with intellectual disability and so were not relevant for our purpose.

Given that our systematic search failed to identify any articles on this topic, we believe there is a need for a brief overview of long‐COVID and how it may relate to adults with intellectual disabilities. Due to the lack of data, we can only make extrapolations from what is known about the condition within the general population. Our aim is to highlight areas for practice and future research from a psychosocial perspective. We have focused specifically on signs of long‐COVID, prevalence, risk factors and treatment of long‐COVID in this population. We recognise this is not an exhaustive review, which is partly reflective of the state of the literature but also of the author's area of speciality being clinical psychology.

Evidence has already shown differences in how adults and children have been affected by COVID‐19 (National Institute for Health and Care Excellence, Scottish Intercollegiate Guidelines Network & Royal College of General Practitioners, 2022), not to mention the disparities in needs, support systems and healthcare provision between the two groups. Therefore, we feel a separate dedicated review of long‐COVID in children with an intellectual disability is warranted.

### 1.1 Signs and symptoms of long‐COVID

Symptoms of long‐COVID within the general population can vary. This has been demonstrated by a meta‐analysis of 15 studies, which found more than 50 long‐term effects of COVID‐19 as self‐reported by individuals recovering from the virus (Lopez‐Leon et al., 2021). Presenting as a heterogenous condition (Michelen et al., 2021), common symptoms may include pain, weakness, fatigue, dyspnoea (shortness of breath), prolonged coughing, sleep disturbances, cognitive impairment such as ‘brain fog’ or attention disorder, hair loss and ear, nose and throat symptoms. It is also important to recognise that psychological difficulties can be commonly experienced in those with the condition, such as anxiety and depression, social isolation, posttraumatic stress disorder and obsessive‐compulsive disorder (Lopez‐Leon et al., 2021). At this stage, it is unknown whether the condition may present as a range of clinical syndromes characterised by specific clusters of symptoms (Michelen et al., 2021).

There is some evidence to suggest certain symptoms, such as fatigue, breathing difficulties or coughing, sleep difficulties, anxiety and depression, hair loss and cognitive impairment may be more frequently reported symptoms of long‐COVID within the general population (Chen et al., 2022); however, the prevalence of symptoms tends to differ between studies. Severity and course of symptoms can vary, with a persistent, relapsing or remitting pattern (Nabavi, 2020). For obvious reasons, there is a lack of evidence concerning the longitudinal manifestations of the condition—this may also be accounted for by the limited systematic reporting or data collected from patient follow‐up (Garg et al., 2021).

Studies investigating symptoms of long‐COVID within the general population have typically involved asking patients who have recovered from acute COVID‐19 to volunteer to complete specifically designed questionnaires or surveys (Garg et al., 2021). However, as in other conditions that are investigated via subjective accounts, this approach may pose a number of difficulties for people with an intellectual disability who may have limited insight into their current and pre‐COVID‐19 health status, a lack of understanding—which may be further impaired by long‐COVID related cognitive difficulties, and challenges in communication.

A concern is that people with an intellectual disability are suffering with long‐COVID in silence, as we fail to identify the symptoms or recognise how the syndrome presents in this group—and subsequently, patients are not referred on to receive appropriate care. In fact, in July 2022, NHS England (2022) reported on average 1500 people are referred to post‐COVID services each week, with people with learning disabilities being underrepresented and services seeing very few individuals. While the cause of this is still unclear, adults in the general population have also described experiencing problems when presenting to services for support with long‐COVID, such as feeling dismissed, ignored or being misdiagnosed (Macpherson et al., 2022). Failing to recognise the additional needs of people with an intellectual disability and long‐COVID may cause additional barriers when seeking care, which may only further existing inequalities. There is likely a need for existing screening tools, surveys and questionnaires for assessing and monitoring long‐COVID to be adapted, as well as new ones to be developed, including measures that gather data from caregivers or informants. Such tools may focus more on behavioural or observable symptoms of long‐COVID and be specific to how the condition presents in this population. For instance, research has shown differences in acute COVID‐19‐related symptoms, with people with an intellectual disability being less likely to report a loss of taste and smell, compared to the general population (Heslop et al., 2021). There is evidence to suggest long‐COVID can be associated with organic‐system‐specific injuries which may be investigated using pulmonary, cardiovascular, cutaneous, musculoskeletal and neuropsychological tests (Garg et al., 2021). This suggests a possible role for objective measures to identify or monitor long‐COVID in people with an intellectual disability. This approach would be consistent with current treatment guidelines for long‐COVID in England, suggesting cognitive, psychological and physical factors should be examined.

Finally, diagnostic overshadowing is also an obvious concern, as individuals may be at risk of the detrimental impact of COVID‐19 being erroneously associated with other comorbidities or overlooked altogether, as they may present with atypical symptoms. Clinical assessments, screening measures—ideally delivered in a face‐to‐face setting for administers to obtain qualitative data—and a review of previous clinical presentations should all be considered when assessing for the condition in people with an intellectual disability. This is particularly important when examining the degree of recovery, as factors to indicate improvement from long‐COVID may differ between individuals with an intellectual disability and the general population. Indeed, qualitative approaches to researching long‐COVID in this group are likely to be invaluable in helping to develop a nuanced insight and to inform future larger scale studies—although as of yet, the lived perspectives of this group have unfortunately been underrepresented (Doody & Keenan, 2021). Patients' accounts of the condition may help to identify key questions to ask in assessments or common difficulties to target in treatment.

### 1.2 Prevalence

A systematic review of the literature performed in March 2022 analysed studies investigating rates of long‐COVID symptoms in people 28 days or more following infection of COVID‐19. The review included 50 studies investigating the data from almost 1.7 million individuals. A pooled prevalence rate of 0.43 (43%) was found. The authors helpfully put this into context, explaining best estimates suggest 470 million people have been infected with the virus worldwide, thus meaning, approximately 200 million will have experienced long‐term health consequences of COVID‐19 (Chen et al., 2022). In the United Kingdom more specifically, in March 2022, the Office for National Statistics (2022) estimated 2.4% of the UK population experienced post‐COVID symptoms 4 weeks or more after suspecting they had contracted the COVID‐19 virus.

While there has been an ever‐increasing number of studies investigating the prevalence of long COVID, we have been unable to find any research ascertaining the rates in people with an intellectual disability. While a subgroup of the samples investigated in the studies so far may have included adults with an intellectual disability, authors fail to report this information (or whether they have been excluded and for what reason) or stratify their findings based on a diagnosis of an intellectual disability. It may be possible for existing studies in this area to conduct secondary analyses of data sets examining rates of long‐COVID in this group specifically. Population based studies with a sufficient sample size would be most appropriate for this aim as analyses could also be performed to identify and control for covariates such as demographic, social support and socioeconomic factors (Totsika et al., 2021).

The global prevalence of intellectual disabilities is believed to be approximately 1% (Maulik et al., 2013), therefore it is reasonable to suggest a substantial number of people with an intellectual disability will also be living with the long‐term effects of the virus. Alternatively, if the lack of research on long‐COVID in people with an intellectual disability reflects a true low prevalence in this group, it may have important implications for how we understand long‐COVID in people without an intellectual disability.

A clear priority in this area is to better understand the prevalence of long‐COVID in people with an intellectual disability. Such research could be undertaken in a range of ways at the local, national and international level. For example, in England, many local authorities and most General Practitioners hold registers of their residents or patients who have an intellectual disability—some services may have also kept a record of patients who have been diagnosed with COVID that has been used to inform care plans and follow‐up. Thus, it would not be difficult to access information on their physical and psychological symptoms or their experiences through carrying out audits or a research exercise into the prevalence, nature and impact of long‐COVID on their lives. Other methods may include conducting cross‐sectional studies by recruiting individuals, family members, professional and nonprofessional carers via healthcare services and national associations for people with intellectual disabilities. It may be particularly helpful to recruit professionals in long‐COVID services to examine the number of people with intellectual disabilities who they have worked with, their experiences of providing care to this group including barriers, facilitators and adaptations to treatment, and existing training and development opportunities specific to intellectual disabilities. A similar study was performed to examine psychiatric care provided to this group across several NHS services during the lockdown (Rauf et al., 2021).

### 1.3 Risk factors for long‐COVID

It is beyond the scope of this article to discuss the aetiology of long‐COVID; however, it is important to note that proposed causes can be multifactorial and not always known or agreed upon (Michelen et al., 2021). A recent review of long‐COVID outlines several possible pathophysiological mechanisms which can be mapped on to the acute infection, inflammatory response and recovery phases of the virus. Organ damage, inflammation, immune response, effect of treatments, complications associated with the virus, interaction with comorbidities, psychological factors, deconditioning and social and financial impact are some of the factors that have been proposed (see Raveendran et al., 2021).

Identifying groups at high risk for severe outcomes from COVID‐19 has been highlighted as an important objective and can help to inform preventative measures and treatment (Williamson et al., 2021). In fact, it was this approach that meant in England, a subgroup of people with an intellectual disability were prioritised for the COVID‐19 vaccination (Public Health England, 2021). Risk factors for long‐COVID identified within the general population may help us to make several inferences on why people with an intellectual disability could be at a greater risk of continuing to experience ongoing symptoms after the acute infection.

The first factor to note is that people must have been infected with the COVID‐19 virus to experience long‐COVID. As previously discussed, studies around the world have identified higher rates of the infection in people with an intellectual disability. Nevertheless, as COVID‐19 can be asymptomatic, because of false negative test results and access to tests may have been low for this group (Doody & Keenan, 2021), simply relying on whether individuals with an intellectual disability were diagnosed with COVID‐19 or not as an indicator, is not suitable to rule out the possibility of long‐COVID.

Other possible risk factors for experiencing symptoms 4 weeks or longer following acute COVID‐19 in the general population include sex (with females being more at risk), ethnicity, poor pre‐pandemic mental and general health, pre‐pandemic long‐term health conditions such as asthma, overweight or obesity and smoking or vaping. However, it is important to recognise that the quality of research in this area has been assessed as low and at risk of bias (National Institute for Health and Care Excellence, Scottish Intercollegiate Guidelines Network & Royal College of General Practitioners, 2022). Notwithstanding such criticisms, it is well known that individuals with an intellectual disability are at a greater risk of experiencing mental health difficulties and other medical comorbidities (Smiley, 2005), which possibly places them at a greater risk—particularly in the context of proposed causes of long‐COVID outlined above. In fact, it has been suggested that the increased likelihood of preexisting comorbidities in this group has meant they are more likely to experience severe outcomes associated with the virus resulting in some of the inequality in mortality and morbidity rates (Doody & Keenan, 2021). Moreover, evidence collected in the United Kingdom suggests long‐COVID is more common in those with another activity‐limiting health condition or disability, and in people living in more deprived areas (Office for National Statistics, 2022). Arguably, these are characteristics commonly seen in people with an intellectual disability.

The severity of acute COVID‐19 may also pose an increased risk, with evidence suggesting previously hospitalised individuals are more likely to experience persistent symptoms following their recovery from COVID‐19 (Iwua et al., 2021). However, there is also evidence to argue that the severity of acute COVID‐19—including whether they were hospitalised—should not be used as a predictive indicator (National Institute for Health and Care Excellence, Scottish Intercollegiate Guidelines Network & Royal College of General Practitioners, 2022). Mixed results may be due to certain groups of individuals such as those who were hospitalised being more likely to be recruited to studies compared to people who have recovered from the virus in the community and did not come into contact with healthcare services (Michelen et al., 2021). It is clear that more high‐quality studies are required to determine the degree to which this is a risk factor, especially given that a cohort study in Canada found people with disabilities (including intellectual disabilities) who were admitted to hospital with COVID‐19 reported poorer outcomes, including longer hospital stays and increased risk of readmissions compared to those without a disability (Brown et al., 2022). Similarly, another study from North America also found individuals with an intellectual disability were more likely to experience severe symptoms of COVID‐19 (Schott et al., 2022).

Research investigating prevalence rates of long‐COVID in people with an intellectual disability could also aim to identify common factors in those experiencing long‐COVID and an intellectual disability compared to those who recovered without long‐term effects. The identification of variables should be informed by significant factors observed in the general population, as well as intellectual disability specific variables, such as level of intellectual disability, healthcare and social needs and living situation with evidence suggesting those living in congregated settings are at a heightened risk of the virus (Doody & Keenan, 2021). Moreover, data has shown that people who have an intellectual disability are nearly 11 times more likely to die prematurely of respiratory disease (Trudale et al., 2021).

### 1.4 Treatment of long‐COVID

In March 2022, the National Institute for Health and Care Excellence, the Scottish Intercollegiate Guidelines Network and the Royal College of General Practitioners produced a document concerning COVID‐19 rapid guidelines for managing the long‐term effects of COVID‐19 (National Institute for Health and Care Excellence, Scottish Intercollegiate Guidelines Network & Royal College of General Practitioners, 2022). In their report, they recognise that there are no internationally agreed clinical definitions or treatment pathways for long‐COVID; however, acknowledged this will be an evolving field as new research and our understanding of the condition improves. The guidelines refer to established treatments for managing symptoms that are associated with long‐COVID, such as support with fatigue, breathing retraining and psychological and psychiatric care. A recommendation is for treatment to be delivered within a multidisciplinary framework with clinical teams including occupational therapists, physiotherapists, clinical psychologists and professionals from psychiatry and rehabilitation medicine. This approach to care should of course also be reflected in research and evidence‐base, recognising the psychological focus of the current article.

Recommendations made in the guidelines have several implications for people with intellectual disabilities. If care of individuals with an intellectual disability and long‐COVID is provided by specialist services, there may be a risk that they have a limited understanding of the individual's pre‐pandemic functioning, thus highlighting the need for a collaborative effort with services sharing medical history and for carers to be involved in patient's care. Professionals in these settings should have access to additional intellectual disability‐specific training, making sure that services are sufficiently adapted to their additional needs. In some cases, cross‐service approaches will likely be required; for example, it is unlikely that professionals working in long‐COVID services will have expertise in managing more complex difficulties commonly associated with intellectual disabilities, including challenging behaviour. Indeed, research has shown how health problems in people with an intellectual disability can contribute to problem behaviours (May & Kennedy, 2017)—and so a similar finding may be found with COVID‐19. For example, possible behavioural symptoms associated with common signs of long‐COVID including fatigue, pain and brain fog could include a reduction in appetite, increase or decrease in sleep, social withdrawal, increase in irritability or anger, greater sedentary behaviour, reluctance to engage in activities, change in mobility and signs of difficulties with concentration such as taking longer to complete activities.

It is likely that services at the primary and community level will act as gatekeepers to refer people to long‐COVID specialists. Therefore, it is essential that all professionals are aware of the signs of long‐COVID in people with an intellectual disability and provide routine screens—particularly as professionals may be more likely to recognise COVID‐19‐related symptoms than family members (Linehan et al., 2022). People with an intellectual disability following acute COVID‐19, may benefit from a follow‐up to help identify symptoms of long‐COVID and any additional care needs and—as recommended by treatment guidelines—should help individuals manage the fear and uncertainty that can be common in those recovering from the virus (Macpherson et al., 2022). Lack of support may only perpetuate (or precipitate) experiences associated with long‐COVID, such as anxiety and depression. Finally, while the benefits of self‐help materials to support adults experiencing long‐COVID are acknowledged, efforts are needed to adapt this literature for people with an intellectual disability making content easy read. We have recently come across such a leaflet produced by Suffolk Learning Disability Partnership (2021), which was posted online in November 2021.

In the guidelines, several considerations are made that are particularly relevant to adults with an intellectual disability. For example, it is suggested that additional support should be provided to people with complex needs, including short‐term and advanced care plans, social and emotional support. It is also recognised that people in vulnerable or high‐risk groups who have self‐managed in the community after acute COVID‐19 may require additional support by services. More specifically however, a key recommendation for research highlighted in the document is to examine differences in treatment effectiveness between the general population and other groups, such as people with an intellectual disability. This is particularly salient as the lack of evidence in this area raises another serious concern about what data is currently being used to inform evidence‐based clinical guidelines to treat adults with an intellectual disability and long‐term effects of COVID‐19. The importance of adapting services for this population in response to challenges associated with pandemic has already been discussed elsewhere (Murray et al., 2021).

There is a growing evidence base supporting the use of psychological interventions for adults with long‐COVID (Department of Health and Social Care, 2021; Kuut et al., 2021); however, the authors are unaware of any research demonstrating the outcome of such treatments in adults with an intellectual disability. This is especially concerning due to the lack of data in adults with intellectual disabilities for the treatment of symptoms such as fatigue, which are commonly reported as part of long‐COVID. For example, while there are strong findings to demonstrate the effectiveness of treatments for comorbid fatigue within the general population (Huizinga et al., 2021; Phyo et al., 2018), very few studies exist investigating fatigue in people with an intellectual disability. The same can be said for other long‐COVID related difficulties, such as pain (Lonchampt et al., 2020) and sleep (Shanahan et al., 2019). Practitioners providing long‐COVID care in this population should look to disseminate their therapeutic approaches and patient‐outcomes. Evidence could be collected across the hierarchy of scientific evidence including funded trials, cohort studies examined as part of service evaluations and single case experimental designs. Indeed, practice‐based research plays an important role in increasing the evidence base for psychological interventions for adults with an intellectual disability. This data can then be used to inform practice and research including cohort studies and clinical trials.

## 2 CONCLUDING REMARKS

While the impact and severity of long‐COVID is well recognised with the NHS investing substantial resources in response (NHS England, 2022), there is a dearth of research involving people with an intellectual disability. This is the first article we are aware of on the condition in this population, as our systemic search failed to identify any relevant studies. The lack of evidence is halting us from developing an understanding of the condition in this population, how people are being affected, and what are the most appropriate forms of treatment. We have approached writing this article with caution and made attempts to not over‐interpret or generalise current data to people with an intellectual disability. It is unacceptable if we are in a similar position in the future, whereby we are looking back on how COVID, in the form of long‐COVID, has widened the inequality in people with an intellectual disability. This article has been written in line with current treatment guidelines on long‐COVID, in that we hope it will raise awareness, and prompt greater sharing of understanding, experiences and findings in this area.

Different methods of scientific inquiry are needed to help identify the prevalence of the condition and how it presents to inform care pathways for assessment, treatment, and prevention of long‐term effects of COVID‐19 in this population. Furthermore, this evidence should be used to educate and inform the workforce and those in training. NHS England (2022) have recently published their plan of the improvement of long‐COVID services, in which they state they are trying to ensure that underrepresented groups, such as those with intellectual disabilities, are able to access services. They recognise that healthcare professionals need to play a role in promoting access to services and so they plan to provide a range of materials that support education and training focusing on the recognition, diagnosis, onward referral and treatment or rehabilitation of those affected. Given the current evidence‐base, there is a worry that such resources will be based on the work and research conducting with the general population. Thus, there is a need to promote in training a space to reflect on how long‐COVID may impact on people with intellectual disabilities, as well as support research in this population.

## CONFLICT OF INTEREST

The authors declare no conflict of interest.

## Data Availability

Data sharing is not applicable to this article as no data sets were generated or analysed during the current study.
